# Epidemiology of Pediatric Trauma and Its Patterns in Western Iran: A Hospital Based Experience

**DOI:** 10.5539/gjhs.v8n6p139

**Published:** 2015-10-26

**Authors:** Fereshteh Jalalvandi, Peyman Arasteh, Roya Safari Faramani, Masoumeh Esmaeilivand

**Affiliations:** 1 Faculty Member of Paramedical School, Kermanshah University of Medical Sciences, Kermanshah, Iran; 2 Non communicable Disease Research Center, Fasa University of Medical Sciences, Fasa, Iran; 3 Student Research Committee, Shiraz University of Medical Sciences, Shiraz, Iran; 4 Department of Biostatistics & Epidemiology, School of Public Health, Kermanshah University of Medical Sciences, Kermanshah, Iran; 5 Faculty Member of Nursing and Midwifery School, Kermanshah University of Medical Sciences, Kermanshah, Iran

**Keywords:** epidemiology, pediatric, childhood, trauma, injury

## Abstract

**Background and Objective:**

Trauma is a major cause of mortality in children aged 1 to 14 years old and its patterns differs from country to country. In this study we investigated the epidemiology and distribution of non-intentional trauma in the pediatric population.

**Materials and Methods:**

The archives of 304 children below 10 years old who presented to Taleghani trauma care center in Kermanshah, Iran from March to September 2008, were reviewed. Patients’ demographic and injury related information were registered. The participants were categorized into three age groups of 0-2, 3-6 and 7-10 years old and the data was compared among age groups and between both sexes.

**Findings:**

The most common cause for trauma was falling from heights (65.5%) and road traffic accidents (16.4%). The most common anatomical sites of injury were the upper limbs followed by the head and neck (36.8% and 31.2%, respectively). Injuries mostly occurred in homes (67.4%). The injuries were mostly related to the orthopedics and the neurosurgery division (84.1% and 13.1%, respectively). Accident rates peaked during the hours of 18-24 (41.3%). Male and female patients did display any difference regarding the variables. Children between the ages of 0-2 years old had the highest rate of injury to the head and neck area (40.3%) (p=0.024). Falls and road traffic accidents displayed increasing rates from the ages of 0-2 to 3-6 and decreasing rates to the ages of 7-10 years old (p=0.013). From the ages of 0-2 to 3-6 years old, street accidents increased and household traumas decreased. After that age household trauma rates increased and street accidents decreased (p=0.005). Children between the ages of 7-10 years old had the highest rate of orthopedic injury (p=0.029).

**Conclusion:**

Special planning and health policies are needed to prevent road accidents especially in children between the ages of 3-6 years old. Since homes were the place where children between the ages of 0-2 were mostly injured, parents should be educated about the correct safety measures that they need to take regarding their children’s environments. The orthopedics department needs to receive the most training and resources for the management of pediatric trauma.

## 1. Introduction

Trauma is defined as any type of wound or penetrating/non-penetrating injury caused intentionally or unintentionally by external factors and this includes a variety of events like traffic-related trauma, poisoning, falling, drowning, and etc. (Partrick, Bensard, Moore, Partington, & Karrer, 1998; Schneiderman, Leslie, Hurlburt, Zhang, & Horwitz, 2012). Trauma is the most prevalent cause of death in the first three decades of life and studies conducted show that it is among the major causes of mortality, especially in children between the ages of 1 to 14 years old (Pant, Towner, Pilkington, Ellis, & Manandhar, 2014). Over 80% of all 875.000 deaths that occur annually in children under 18 years old worldwide happen in countries with low and middle income (Peden, 2008). Permanent disabilities that results from these accidents in children causes prolonged disability periods and imposes a huge mental and financial burden on the child, family and the society (Alexandrescu, O’Brien, & Lecky, 2009; Peden, 2008). In Europe studies have shown that these injuries are responsible for about 19% of the disability adjusted life years (DALYs) for children and adolescence between the ages of 0-19 years old (Simon, Gilyoma, Dass, McHembe, & Chalya, 2013). Children bellow the ages of ten years old are especially at higher risks of accidents due to multiple factors like their inability to react and recognize danger in time and their impaired risk assessment (Cross & Hall, 2005).

The epidemiology and pattern of trauma related accidents in the pediatric population differ from one country to another based on variables like the socio-economic status, the geographical and population related characteristics of that particular region (Chabok, Ramezani, Kouchakinejad, & Saneei, 2012; de Sousa Petersburgo et al., 2010; Durkin, Olsen, Barlow, Virella, & Connolly, 1998; Edwards, Green, Lachowycz, Grundy, & Roberts, 2008).

Considering the fact that the majority of the trauma accidents are preventable, especially in the pediatric population, understanding the pattern of trauma in every region is necessary for health related policy making and preventive measures (Cross & Hall, 2005; Densmore, Lim, Oldham, & Guice, 2006).

In this study we investigated the epidemiology of non-intentional trauma regarding etiology spectrum, location of trauma, anatomical site of injury and distribution of trauma based on different factors in children bellow ten years old.

## 2. Materials and Methods

### 2.1 Study Setting

This cross-sectional study was conducted in Taleghani trauma center in Kermanshah, Iran. The archives of all trauma cases that referred to our care center and were below the ages of 10 years old, from March 2008 to September 2008, were reviewed. The study protocol was approved by both the Ethics Committee and the institutional review board of Kermanshah University of Medical Sciences. Secrecy of patients’ information was maintained throughout the study and no data regarding patients’ personal information was disclosed. Our medical care center is one of the most important referral trauma centers in the west of Iran and the majority of trauma patients from the neighboring provinces refer to our center to receive medical services.

### 2.2 Study Protocol

For determining the study sample, 3 days were selected randomly in each month using random numbers and the patients’ records that referred on that day were included in the study. Any records that had missing data included self-inflicted or intentional injuries and if the child did not have any parents or guardians, were excluded from the study.

Finally 304 cases were evaluated. Patients’ data were extracted using a checklist that included: demographic information, cause of trauma, anatomical site of injury, location in which the trauma had occurred, the department which the trauma related to (considered as the type of injury) and the time of day in which the trauma had occurred.

For better comparison we categorized the participants into three age groups of 0-2, 3-6 and 7-10 years old and the data was compared among age groups. Injury related data was also compared between males and females.

### 2.3 Statistical Analysis

The analysis of the data was done using the Statistical Package for Social Sciences software, SPSS for windows, version 16 (SPSS Inc., Chicago, IL, USA). For comparison of data between the groups the independent t-test and the chi-square test were used for quantitative and qualitative data, respectively. Data are displayed as frequency, percent, means and standard deviations. A two-tailed p-value of less than 0.05 was considered as statistically significant.

## 3. Results

About 58.9% out of the 304 children were males. The most common cause for trauma for both males and females was falling from heights (65.5% of overall cases, 71.2% in girls and 61.5% in boys) and traffic-related accidents (16.4%).

The most common anatomical sites of injury were the upper limb, head and neck and lower limbs, respectively (36.8%, 31.2% and 16.8%).

Regarding the setting of the accidents, they mostly occurred in homes (67.4%). Evaluating the type of injury based on the department which the injuries were referred to, showed that the majority of the injuries were related to the orthopedics division followed by the neurosurgery division (84.1% and 13.1%, respectively). Accidents mostly occurred afternoon, peaking during the hours of 18-24 (41.3%).

Although male patients had higher rates of multiple trauma (15.1% vs. 4.8%) and females displayed higher rates of upper extremity and head and neck injuries (39.2% vs. 35.2 and 34.4 vs. 29.1, respectively), overall there was no significant difference between the two sexes regarding site of injury (p = 0.122).

A higher percent of girls were injured in their homes compared to boys (72% vs. 64.2%), meanwhile males had an overall higher rate of trauma outside their homes compared to females. Males and females did not display a significant difference regarding their setting of trauma (p = 0.68).

There was no difference between male and female patients regarding mechanism of injury, type and time of injury (p = 0.404, p = 0.813 and p = 0.766, respectively) (Table 1).

**Table 1:** Trauma related characteristics among male and female patients

Variables	Male (n= 179)	Female (n= 125)	Overall	p-value	Variables	Male (n= 179)	Female (n= 125)	Overall	p-value
Location of injury - no. (%)					Place of trauma - no. (%)				
Upper limb	63 (35.2)	49 (39.2)	112(36.8)		Home	115 (64.2)	90 (72)	205 (67.4)	
Head & neck	52 (29.1)	43 (34.4)	95 (31.2)		Street	54 (30.2)	31 (24.8)	85 (28)	
Lower limb	30 (16.8)	21 (16.8)	51 (16.8)	0.122	Highways	4 (2.2)	2 (1.6)	6 (2)	0.68
Multiple trauma	27 (15.1)	6 (4.8)	33 (10.9)		School	5 (2.8)	2(1.6)	7 (2.3)	
Jaw	3 (1.7)	2 (1.6)	5 (1.6)		Other	1 (0.6)	0 (0)	1 (0.3)	
Hip & abdomen	2 (1.1)	2 (1.6)	4 (1.3)		Total	179 (100)	125 (100)	304 (100)	
Spine	1 (0.6)	2 (1.6)	3 (1)						
Chest	1 (0.6)	0	1 (0.3)						
Total	179 (100)	125 (100)	304 (100)						

Mechanism - no. (%)					Related department of injury* - no. (%)				

Falling	110 (61.5)	89 (71.2)	199(65.5)		orthopedics	74 (83.1)	48 (85.7)	122(84.1)	
Road traffic accidents	32 (17.9)	18 (14.4)	50 (16.4)		Neurosurgery	12 (13.5)	7 (12.5)	19 (13.1)	
Collision with objects	15 (8.4)	6 (4.8)	21 (6.9)		General surgery	2 (2.2)	1 (1.8)	3 (2.1)	0.813
Cuts	8 (4.5)	6 (4.8)	14 (4.6)		Outpatient	1 (1.1)	0 (0)	1 (0.7)	
Falling objects	3 (1.7)	3 (2.4)	6 (2)	0.404	Total	89 (100)	56 (100)	145 (100)	
Dispute	2 (1.1)	0 (0)	2 (0.7)		Time of day¶				
Mines	2 (1.1)	0 (0)	2 (0.7)		0 – 6	11 (6.1)	5 (4)	16 (5.3)	
Animal bites	1 (0.6)	0 (0)	1 (0.3)		6 – 12	38 (21.2)	28 (22.6)	66 (21.8)	
Exercise trauma	0 (0)	1 (0.8)	1 (0.3)		12 – 18	54 (30.2)	42 (33.9)	96 (31.7)	0.766
Other	6 (3.4)	2 (1.6)	8 (2.6)		18 – 24	76 (42.5)	49 (39.5)	125(41.3)	

Total	179 (100)	125 (100)	304 (100)		Total	179 (100)	124 (100)	303 (100)	

* Outpatient was all those who did not need to be referred to any of the other departments and the information of 159 patients were missing in the patients’ records.

¶ Information of one patient was missing.

When we compared the data among the different age groups, our results showed that children between the ages of 3-6 had the highest rates of accidents compared to the other age groups (39.7%, 34% and 26.1% for children in the ages of 3-6, 7-10 and 0-2, respectively).

Children between the ages of 0-2 years old had the highest rate of injury to the head and neck area (40.3%) while this was less in older children (30.6% in children between 7-10). Contrary to the rate of injuries to the head and neck, injuries to the extremities were highest in older children. Overall the age groups displayed a significant difference regarding their anatomical site of injury (p = 0.024).

Regarding the etiology of trauma, falls and road traffic accidents displayed increasing rates from the ages of 0-2 to 3-6 (from 75.3% to 64.1% and from 9.1% to 23.1%, for falling and traffic accidents, respectively). After which they displayed subsequent decreased rates to the ages of 7-10 years old (from 64.1% to 60% and from 23.1% to 15% for falls and road accidents, respectively). The three age groups displayed a meaningful difference in their mechanism of trauma (p = 0.013).

From the ages of 0-2 to 3-6 years old, trauma rates occurring in the streets increased and those occurring in the setting of households decreased. After that age household trauma increased and street accidents decreased. Overall the age groups showed a meaningful difference regarding their location of trauma (p = 0.005).

Regarding type of injury, orthopedics related trauma had the highest number of patients in all age groups. Overall the types of injury were significantly different among the three age groups (p = 0.029).

Although the age groups did not display a significant difference regarding their time of trauma (p = 0.97), children between the ages of 0-2 had the highest rates of trauma during the hours of 18-24 (44.2%) and children between the ages of 3-6 years old had the highest rates of trauma during the hours of 12-18 (34.5%) (Table 2).

**Table 2:** Trauma related characteristics among different age groups

Variables	0 - 2	3 - 6	7 - 10	Overall	p-value	Variables	0 - 2	3 - 6	7 - 10	Overall	p-value
Location of injury*						Area of trauma*					
Head & neck	31 (40.3)	36 (30.8)	23 (23)	90 (30.6)		Home	63 (81.8)	71 (60.7)	63 (63)	197 (67)	
Upper limb	25 (32.5)	38 (32.5)	46 (46)	109 (37.1)		Street	13 (16.9)	45 (38.5)	26 (26)	84 (28.6)	
Lower limb	14 (18.2)	17 (14.5)	20 (20)	51 (17.3)		Highways	0	0	5 (5)	5 (1.7)	
Multiple trauma	6 (7.8)	17 (14.5)	8 (8)	31 (10.5)	0.024	School	1 (1.3)	0	6 (6)	7 (2.4)	0.005
Jaw	0	3 (2.6)	2 (2)	5 (1.7)		Other	0	1 (0.9)	0	1 (0.3)	
Hip & abdomen	1 (1.3)	3 (2.6)	0	4 (1.4)		Total	77 (100)	117 (100)	100 (100)	294 (100)	
Spine	0	2 (1.7)	1 (1)	3 (1)							
Chest	0	1 (0.9)	0	1 (0.3)							
Total	77 (100)	117 (100)	100 (100)	294 (100)							
Mechanism*						Related department for injury¶					
Falling	58 (75.3)	75 (64.1)	60 (60)	193 (65.6)		orthopedics	25 (83.3)	50 (76.9)	46 (93.9)	121 (84)	
Road traffic accidents	7 (9.1)	27 (23.1)	15 (15)	49 (16.7)		General surgery	0	3 (4.6)	0	3 (2.1)	
Collision with objects	7 (9.1)	7(6)	5 (5)	19 (6.5)	0.013	Neurosurgery	5 (16.7)	11 (16.9)	3 (6.1)	19 (13.2)	0.029
Cuts	2 (2.6)	3 (2.6)	9 (9)	14 (4.8)		Outpatient	0	1 (1.5)	0	1 (0.7)	
Falling objects	0	2 (1.7)	3 (3)	5 (1.7)		Total	30 (100)	65 (100)	49 (100)	144 (100)	
Dispute	0	0	2 (2)	2 (0.7)		Time of day¥					
Mines	0	0	2 (2)	2 (0.7)		0-6	3 (3.9)	7 (6)	5 (5)	15 (5.1)
Animal bites	0	1 (0.9)	0	1 (0.3)		6-12	19 (24.7)	22 (19)	22 (22)	63 (21.5)	
Exercise trauma	0	1 (0.9)	0	1 (0.3)		12-18	21 (27.3)	40 (34.5)	32 (32)	93 (31.7)	0.978
Other	3 (3.9)	1 (0.9)	4 (4)	8 (2.7)		18-24	34 (44.2)	47 (40.5)	41 (41)	122 (41.6)	
Total	77 (100)	117 (100)	100 (100)	294 (100)		Total	77 (100)	116 (100)	100 (100)	293 (100)	

* Information of ten patients was missing.

¶ Information of 160 patients was missing with regards to the age groups.

¥ Information of 11 patients was missing with regards to the age groups.

## 4. Discussion

The findings of the present study showed that the prevalence of pediatric trauma in boys exceeds trauma rates in girls, though the difference was not significant, it was similar to the findings of Mutto et al. (Mutto, Lawoko, Nansamba, Ovuga, & Svanstrom, 2011) in 2011, where they evaluated the pattern and odds of trauma in 566 children bellow the ages of 13 years old in Kampala city. They found that almost 60% of patients were males. This was also seen in the study by Zia et al. (Zia, Khan, Razzak, Puvanachandra, & Hyder, 2012) in 2012, where they documented that 66% of the injured children (bellow 12 years old), were males. This finding was also seen in other studies (Isaac, Van Niekerk, & Van As, 2014). This could be due multiple factors including that boys have more freedom to play in different developing countries and boys put themselves in more dangerous situations compared to girls because of their sex related characteristics.

Regarding the etiology of pediatric trauma our results showed that falling from heights is the leading cause of trauma in both sexes and traffic accidents was the second cause in Iran. In the study by Herbert et al. (2012) evaluating pediatric injuries during the years of 1997 to 2006 in South Africa they found that the most common cause of trauma was falls (39.8%), followed by road traffic injuries (15.7%) which was similar to our study. Zia et al. (2012) also reported falls as the most common etiology of pediatric trauma (59%) in Karachi, although the second cause in their study was dog bites (11%) and road traffic accidents was the fourth cause of injury (6%). This is probably due to the economic and environmental differences between the countries.

The results of the present study show that trauma to the upper extremities is the most prevalent anatomic site of injury in both sexes. Studies have shown different results regarding this matter like in the study by Simon et al. (2013) in Tanzania, they found that injuries to the head and neck were the most prevalent type of trauma in children bellow the ages of ten years old (32.7%), meanwhile in the study by Petridou et al. in Greece, they documented that the extremities were the most common site of injury in children between the ages of 0-14 years old (55.3%).

Mayer et al. (Mayer, Walker, Johnson, & Matlak, 1981) also showed that trauma to the head is the most common type of injury in the pediatric population (78.8%) (Mayer et al., 1981). It seems that this difference could be in direct relationship with some factors like age which predisposes children to some injuries. This predisposition is caused by special events and characteristics which is attributed to children in s specific age for example as children become older, in their school ages they are at higher risks of road accidents, which according to some studies (Zafarghandi, Modaghegh, & Roudsari, 2003), subsequently increases head and face injury rates. In one study assessing traffic accidents in children (Zafarghandi et al., 2003), they found that injuries to the face and head was the most prevalent type of injury regarding road accidents. Each study also had different population sizes in the different age groups as a cause to the difference among the studies. Our results also found a relationship between age and anatomical location of injury (p = 0.024). This relationship was also seen in other studies (MacInnes & Stone, 2008).

Between the hours of 18-24, was the time when the majority of the accidents had occurred. If we consider day time between the hours of 6 to 18, this was the time which the majority of the trauma had occurred (53.5%), which was also seen in the study by Simon et al. (Simon et al., 2013) where 83.3% of the trauma had occurred during day time. Our rate of trauma during day time was less than that documented in the mentioned study. This could have been due to the differences in the pattern of injuries in the two studies, whereas the majority of the trauma in their study was due to road traffic accident which, for children bellow the ages of 10 mostly occurs during the day and attributed to their high rates of injuries during the day time.

Our results showed that the most common location of trauma for both sexes was in homes (67.4%). In the study by Maclnnes et al. (MacInnes & Stone, 2008) they evaluated children bellow the ages of 7, and found that homes were the first most common location of injuries followed by footpaths and roads (85%, 3.7% and 1.8%, respectively).

While Mutto (Mutto et al., 2011) found schools to be second most common location of injury, in our study schools were at fourth place. The reason for this difference might have been due to the age limit in our study (bellow 10 years old) and that our study had fewer school pupils compared to the mentioned study (bellow 13 years old).

Mclness et al. (MacInnes & Stone, 2008) found that as children age the pattern of injury changes and with an increase in age, injuries to the head show a subsequent decrease while injuries to the extremities increase. This finding was similar to that documented in our study. This could be due to the fact that as children grow, they get more control over their extremities thus changing their type of activity and pattern of injury.

This pattern was also seen regarding the etiology of injury and with increased age we saw a decrease in the rates of fall injuries and collision injuries, while injuries due to traffic accident and cuts increased. The same pattern was seen in the study by Mclness and colleagues (MacInnes & Stone, 2008) regarding falls but not with collisions. This could be because they studied children less than 7 years old, while we studied children up to 10 years old and we had a wider spectrum of age groups which could have affected the trend of changes. This was not seen in the study by Zia et al. (2012), where fall rates increased to 11 years old. This could have been due to the environmental circumstances and life style differences in Karachi, Pakistan that predisposes children to higher risks of falls.

Our study showed that injuries in homes decreased by increased age and injuries in other areas increases which is expected since children, as they grow discover new environments and spend more time outside the house.

We also found that with increased age neurosurgical related trauma decreased, while orthopedics trauma increased. This might have been due to the fact that children as they become older they become more aware of their environment and with a decrease in injuries to the head and neck, neurosurgical related trauma also decrease.

Our findings indicate that special planning and health policies are needed to prevent road accidents especially in children between the ages of 3-6 years old, when children are at an age where they learn how to walk independently and are faced with a new environment putting them at higher risks of road accidents.

Since homes were the place where children between the ages of 0-2 were mostly injured, parents should be educated regarding the correct safety measures that they need to take regarding their children’s environment. In different age groups the orthopedics department was the most referred division, so this department needs to receive the most training and resources for the management of pediatric trauma.

This study had some limitation, first of all was its retrospective nature which did not give us complete control over the variables, secondly we used the departments which the injuries were referred to as an indicator of the type of injury which is not very precise and different departments might have had an overlap in some cases. The study had some strength as it was among the very few epidemiologic studies evaluating pediatric trauma in the region.

Studies in a wider scale need to be conducted to evaluate and compare the epidemiologic patterns between developing and developed countries, to give a better understanding of the health related programs that are needed in these countries.
